# Recommendations for the development of erosion‐resistant tidal marshes to enhance nature‐based flood risk mitigation

**DOI:** 10.1002/eap.70306

**Published:** 2026-09-22

**Authors:** Marte M. Stoorvogel, Stijn Temmerman, Bas W. Borsje, Victoria G. Mason, Pim W. J. M. Willemsen, Ken Schoutens, Laura Airoldi, Christine Angelini, Jim van Belzen, Gregory S. Fivash, Angus Garbutt, Qiang He, Tjisse van der Heide, Zhan Hu, Johan van de Koppel, Iris Möller, Lotte Oosterlee, Brian R. Silliman, Martin W. Skov, Carter S. Smith, Zhenchang Zhu, Tjeerd J. Bouma

**Affiliations:** ^1^ Department of Estuarine and Delta Systems NIOZ Royal Netherlands Institute for Sea Research Yerseke The Netherlands; ^2^ Department of Physical Geography Utrecht University Utrecht The Netherlands; ^3^ Water Engineering & Management University of Twente Enschede The Netherlands; ^4^ Ecosphere, Department of Biology University of Antwerp Antwerp Belgium; ^5^ Hydrology and Environmental Hydraulics Group, Department of Environmental Sciences Wageningen University and Research Wageningen The Netherlands; ^6^ Department of Ecosystems and Sediment Dynamics Deltares Delft The Netherlands; ^7^ Hydrobiological Station “Umberto D'Ancona”, Department of Biology University of Padova, Uo CoNISMa Chioggia Italy; ^8^ National Biodiversity Future Center Palermo Italy; ^9^ Department of Environmental Engineering Sciences, Engineering School for Sustainable Infrastructure and Environment University of Florida Gainesville Florida USA; ^10^ Wageningen Marine Research Yerseke The Netherlands; ^11^ Centre for Ecology and Hydrology Bangor UK; ^12^ Coastal Ecology Lab, MOE Key Laboratory for Biodiversity Science and Ecological Engineering School of Life Sciences, Fudan University Shanghai China; ^13^ Department of Coastal Systems NIOZ Royal Netherlands Institute for Sea Research Den Burg The Netherlands; ^14^ Conservation Ecology Group, Groningen Institute for Evolutionary Life Sciences University of Groningen Groningen The Netherlands; ^15^ School of Marine Sciences, Sun Yat‐Sen University and Southern Marine Science and Engineering Guangdong Laboratory (Zhuhai) Zhuhai China; ^16^ Guangdong Provincial Key Laboratory of Marine Resources and Coastal Engineering Guangzhou China; ^17^ Pearl River Estuary Marine Ecosystem Research Station, Ministry of Education Zhuhai China; ^18^ Department of Geography Trinity College Dublin Dublin Ireland; ^19^ Nicholas School of the Environment, Duke University Durham North Carolina USA; ^20^ School of Ocean Sciences, Bangor University Bangor UK; ^21^ School of Aquatic and Fishery Sciences, University of Washington Seattle WA USA; ^22^ Guangdong Basic Research Center of Excellence for Ecological Security and Green Development, Guangdong Provincial Key Laboratory of Water Quality Improvement and Ecological Restoration for Watersheds Guangdong University of Technology Guangzhou China

**Keywords:** coastal resilience, nature‐based flood defense, nature‐based solutions, sediment stability, tidal marsh restoration, tidal marsh vegetation

## Abstract

There is growing interest in nature‐based hybrid flood risk mitigation, in which wetland ecosystems, such as tidal marshes, are an integral part. Tidal marshes can attenuate wave loading on defense structures, reduce breach depth in case of defense structure failure, attenuate storm surges, reduce shoreline erosion, and grow vertically with sea‐level rise. Marshes are currently being restored and created for flood risk mitigation and/or for other ecosystem services, making it crucial that they can persist in the long term and therefore develop erosion‐resistant sediment beds. In this paper, we first discuss the key physical and biological processes and properties that drive the development of erosion‐resistant sediment beds in tidal marshes, showing that vegetated, consolidated sediment beds, effectively functioning food webs, and a wide intertidal zone with a variety of ecosystems are highly important for the realization of marsh restoration/creation projects for flood risk mitigation. Second, we discuss how the location (i.e., either seaward or landward of a defense structure) and the physical conditions (i.e., elevation relative to the tidal range and the strength of local wave forcing) under which tidal marsh restoration/creation takes place can determine which interventions will be most suitable. Possible interventions include: sediment nourishment, stabilization structures, managed realignment, transitional polder, regulated tidal exchange, vegetation planting/transplanting, and/or creek excavation. The location and the chosen restoration or creation intervention will strongly affect how fast marsh establishment and erosion resistance development will occur. Overall, our study reveals that successful marsh restoration/creation for nature‐based flood risk mitigation requires (1) selecting the most appropriate marsh restoration or creation intervention for a specific location, and (2) giving the marsh enough time to develop before it is needed for flood risk mitigation. The incorporation of marshes within nature‐based flood risk mitigation strategies should not only be considered at an individual site scale but also upscaled to large landscape scale consequences for flood risk mitigation, to ensure a fully functioning wider ecosystem with good connectivity between ecosystems.

## INTRODUCTION

Coastal and estuarine flood risks are globally increasing due to land subsidence and climate change effects, such as sea‐level rise and locally increasing frequency and intensity of extreme weather events (He & Silliman, 2019; IPCC, 2023; Kirezci et al., 2020; Syvitski et al., 2009). Flooding within coastal and estuarine regions affects many people, given the high population densities (Neumann et al., 2015; Paprotny et al., 2018). Accordingly, coastal regions are often protected by artificially constructed—so‐called gray—flood defense structures (referred to as embankments, dikes, seawalls, levees), for which maintenance is expensive and labor‐intensive in the long term, especially considering effects of current climate change (Temmerman et al., 2013; Zelst et al., 2021). There is growing interest in nature‐based—so‐called green—flood risk mitigation, where wetland ecosystems like tidal marshes, mangrove forests, seagrass meadows, oyster beds, and mussel reefs contribute to flood risk mitigation strategies (Barbier et al., 2011; Bouma et al., 2014; Schoonees et al., 2019; Temmerman et al., 2023). These nature‐based approaches often need to be combined with artificial flood defense structures and are then referred to as “green‐gray” or hybrid solutions (Schoonees et al., 2019). However, wetlands in many places are deteriorating (Airoldi & Beck, 2007; Silliman et al., 2009) and between 2000 and 2019, 1453 km^2^ of salt marshes were lost globally (Brook et al., 2025; Campbell et al., 2022). Salt marshes must therefore be restored (where a marsh has previously been present but was lost or degraded) or created (where a marsh has never existed before) to deliver the intended benefits to communities. Due to the increasing frequency of disturbance events such as storms (IPCC, 2023) and the slow pace of wetland ecosystem establishment following restoration or construction (Gourgue et al., 2022; Zhu, van Belzen, et al., 2020), there is an urgent need to identify optimal methods for allowing the establishment of erosion‐resistant wetland ecosystems for flood risk mitigation within the available time frame.

Tidal marshes are perhaps the best recognized and most studied example of wetland ecosystems that can contribute to flood risk mitigation, which they do in several ways. First, marshes are vegetated and have an elevated bed surface, which results in wave (Möller et al., 2014; Vuik et al., 2016; Willemsen et al., 2020; Zhu, Vuik, et al., 2020) and storm surge attenuation (Stark et al., 2015; Temmerman et al., 2023). Where marshes occur in front of gray defense structures, such as embankments, dikes, seawalls, or levees (from here on “defense structure” is used to refer to all of those gray defense structures), they can reduce both wave impact on these structures and breach probabilities (Vuik et al., 2016; Zhu, Vuik, et al., 2020). Second, if a defense structure breach occurs where the land behind the defense structure (landward) is lower in elevation than the marsh in front of the defense structure (seaward), the elevated bed surface of marshes can reduce water discharge through the breach during the storm surge phase, limit the enlargement of the breach dimensions, and thereby reduce flood damage and casualties in the hinterland (Van den Hoven et al., 2023; Zhu, Vuik, et al., 2020). Third, mature climax marshes can reduce shoreline erosion, by stabilizing sediment beds through their belowground biomass and by promoting sedimentation of fine‐grained cohesive sediments (Brooks et al., 2021; Marin‐Diaz et al., 2022; Schoutens et al., 2022). Additionally, tidal marshes contribute to flood risk mitigation in the long term (decades to centuries) as they are adaptive and climate‐change resilient through two processes. First, if sufficient sediment supply is available, the tidal range is suitable, and sea‐level rise is not too rapid, marshes can keep up relatively well with sea‐level rise due to mineral sediment accretion (Blum et al., 2021; Coleman et al., 2022; Schuerch et al., 2018), as well as organic matter accumulation (Nyman et al., 2006). Second, apart from vegetation, tidal marshes may facilitate the growth of other species communities, such as oyster and mussel reefs (Fivash et al., 2021), which might enhance long‐term flood risk mitigation by increasing bed elevation (Angelini et al., 2015; Crotty et al., 2023) and contributing to wave attenuation (Borsje et al., 2011; Bouma et al., 2014).

For tidal marshes to optimally contribute to long‐term flood risk mitigation, the sediment beds need to be erosion resistant, to not completely erode under wave attacks and under high flow velocities associated with defense structure breaches that may occur over marshes (Schoutens et al., 2022; Zhu, Vuik, et al., 2020). If the marsh erodes laterally or vertically under frequently occurring conditions, during storms, as a result of boat wakes, or after a defense structure breach, the narrowed marsh profile or lowered elevation can reduce the wave attenuation potential or increase discharge through a defense structure breach (Van den Hoven et al., 2023; Vuik et al., 2016; Willemsen et al., 2020), thereby lowering the flood risk mitigation potential of the marsh. This is especially relevant for already narrow marshes (also called fringing marshes) that may completely disappear from small amounts of erosion. However, it is important to note that marshes and adjacent mudflats are dynamic systems that experience natural cycles of lateral and/or vertical erosion and accretion (Bouma et al., 2016; Willemsen et al., 2018). These systems are intrinsically linked to the wider geomorphological setting, which generally provides the conditions of shelter required for depositional landforms. Marsh retreat may, therefore, not immediately decrease their value for flood risk mitigation provided that a marsh of sufficient width and height remains.

Marshes are currently being restored and created for flood risk mitigation and/or other valuable ecosystem services they provide (ABPmer, 2024), such as carbon sequestration (Mason et al., 2023; Temmink et al., 2022); water purification (Nelson & Zavaleta, 2012); habitat provisioning for fish, invertebrates, birds, and others (Bloomfield & Gillanders, 2005; Hughes, 2004; Whitfield, 2017); and recreational activities (Barbier et al., 2011). In this paper, we use the term marsh *restoration* at locations where a marsh has previously been present, but was converted to terrestrial land by land reclamation, lost due to erosion, or degraded, while we use the term marsh *creation* at locations where a marsh has never existed before. Degraded marshes mostly refers to those that have experienced vegetation die‐off, for example due to tidal inundation increases caused by sea‐level rise (Schepers et al., 2017) or due to the combined impact of overgrazing and drought (Silliman et al., 2005). The targeted outcomes of marsh restoration/creation strongly depend on the envisioned ecosystem service contributions of the marsh. For example, when restoring marshes for carbon storage, a high biomass may be an indicator of success, while for habitat provisioning, it may be more important to aim for an ecosystem with a diverse range of vegetation species to create a greater range of niches for fauna. Similarly, the targeted outcomes for a given site may depend on the previous land use, for example whether the site was restored from agricultural land or tidal flats. When restoring or creating marshes for flood risk mitigation or other functions (where flood risk mitigation may be a secondary benefit), it is important that their sediment beds develop erosion resistance, so that they do not erode and become too narrow or too low to significantly contribute to wave and storm surge attenuation, breach depth reduction in case of defense structure failure, and shoreline erosion reduction. The development rate of erosion resistance will depend on the restoration or creation intervention (Stoorvogel, Willemsen, et al., 2025). Hence, depending on the time available before the tidal marsh is needed to fulfill its role in flood risk mitigation, we should choose an approach that creates the conditions that are most likely to lead to the development of erosion‐resistant marsh sediment beds within that time frame. Currently, there are no guidelines to help coastal managers select the most suitable approach, which we aim to address in this paper.

Here, we summarize the key physical and biological processes and properties that drive the development rate of erosion resistance in tidal marshes of relevance to marsh restoration and creation projects (Section: *Drivers of the development rate of marsh erosion resistance*). We further consider how environmental context determines the most suitable restoration or creation interventions, and how contextual conditions and processes will likely affect the development rate of sediment erosion resistance (Section: *Marsh restoration and creation at contrasting locations, possible interventions, and their effect on the development rate of erosion resistance*). We also provide an outlook on the practical implementation of tidal marshes for nature‐based flood risk mitigation and remaining knowledge gaps (Section: *Outlook*).

## DRIVERS OF THE DEVELOPMENT RATE OF MARSH EROSION RESISTANCE

When sediment initially gets deposited on tidal flats or marshes, it is unconsolidated and has a high water content, leading to sediment that is susceptible to erosion (Grabowski et al., 2011). However, sediment erosion resistance will likely develop over time, as a result of several key processes that are discussed in the following sections.

### Sediment accretion

Sediment accretion rates in tidal marshes vary in space and time, as there are many factors that affect sediment accretion rates, such as suspended sediment concentrations and tidal inundation frequency, depth, and duration (Coleman et al., 2022; Fagherazzi et al., 2012; Liu et al., 2021). Sedimentation rates also decrease with distance from tidal creeks and the seaward marsh edge (Reed et al., 1999; Temmerman et al., 2003), indicating the importance of marsh morphology for sediment accretion. Additionally, the occurrence of storms can strongly increase sediment delivery to marshes and thereby sediment accretion (Cahoon, 2006). Such delivery of sediment via storm surges represents a particularly important mechanism of accretion in microtidal marshes (Friedrichs & Perry, 2001; Zhu & Wiberg, 2022), which are generally less resilient to sea‐level rise compared to marshes experiencing a larger tidal range (Kirwan & Guntenspergen, 2010). In turn, sediment accretion rates may affect the development of sediment erosion resistance, as high accretion rates could lead to sediments being buried by new sediment layers before they had time to dry out and consolidate (Brain et al., 2011; Stoorvogel, Temmerman, et al., 2024). The sustainable development of restored and created marshes depends on sediment accretion, although actual development of erosion resistance after sediment is deposited depends on consolidation. Consequently, there is a fine local balance between the rates of sediment accretion and consolidation, as illustrated at the restored marsh of Burchtse Weel (Belgium) for example, where high accretion and low consolidation rates led to very soft and fluid sediment beds (Oosterlee et al., 2020).

Tidal marsh vegetation stimulates the accretion of sediment, both directly by capturing sediment particles with their leaves and stems and indirectly by attenuating hydrodynamic energy which results in suspended sediment settling (Mudd et al., 2010). If these sediment particles are cohesive clays and silts, they will increase cohesion of the sediment bed, and thereby increase erosion resistance (Grabowski et al., 2011). This is a consequence of the mineral composition of silt‐ and clay‐sized particles, which typically consist of a high content of phyllosilicate minerals (so‐called clay minerals like montmorillonite, illite, chlorite, etc.) that have electrostatic charges, causing their cohesive behavior, thereby increasing the cohesive strength and erosion resistance of the sediment bed (Conklin, 2014; Konta, 2009). Sandier marsh sediment beds are more prone to erosion, as cohesive forces are smaller or absent (Feagin et al., 2009; Ford et al., 2016; Lo et al., 2017; Marin‐Diaz et al., 2022). The latter is a consequence of the absence of electrostatic charges due to the mineral composition of sand‐sized particles (consisting of minerals like quartz, feldspar, etc. Conklin, 2014; Konta, 2009). Mostly fine‐grained cohesive sediments are deposited in tidal marshes, as their dense vegetation strongly reduces tidal and wave hydrodynamic forces (Möller et al., 2014; Schoutens et al., 2020), thereby creating a calm environment for fine‐grained cohesive sediment deposition (Stoorvogel, de Smit, et al., 2025; Temmerman et al., 2004). Additionally, higher organic matter contents (both alive and dead organisms and particulate organic matter) of the accreting sediment may increase the cohesive strength and therefore lead to lower erodibility of sediment beds, but this effect strongly depends on the chemical conditions and negative correlation between organic matter content and bulk density (Feagin et al., 2009; Ford et al., 2016; Grabowski et al., 2011). There are also marshes where vertical accretion is not primarily driven by inorganic sediment accretion, but rather by the production and accumulation of organic matter (Nyman et al., 2006). This paper mainly focuses on inorganic sediment accretion, although acknowledging that the relative importance of organic matter accumulation for vertical marsh growth may increase as mineral sediment delivery rates become lower (Peteet et al., 2018; Weston, 2014; Yang et al., 2017).

### Sediment consolidation

Sediment consolidation is an important process in tidal marshes, as it expels pore water from the sediment matrix and makes the sediment bed more densely packed (Brain, 2015). Two types of consolidation that can occur in tidal marshes are (1) self‐weight consolidation and (2) drying‐induced consolidation. Self‐weight consolidation occurs under the weight of new sediments that are deposited on top of existing sediments (Dankers & Winterwerp, 2007; Zhou et al., 2016). Drying‐induced consolidation, on the other hand, occurs when sediment dries out during low tide (Colosimo et al., 2023; Dong et al., 2020; Rees et al., 2024), which may be accelerated by evapotranspiration via vegetation (Barciela‐Rial et al., 2023). The resulting decrease in water content and increase in bulk density lead to increases in sediment strength and erosion resistance (Feagin et al., 2009; Fivash et al., 2024; Grabowski et al., 2011), so generally the faster the consolidation rate, the faster the development rate of marsh erosion resistance. The formation of well‐developed creek networks (Stoorvogel, Temmerman, et al., 2024; van de Vijsel et al., 2023; Van Putte et al., 2022) and micro‐topography (Fivash et al., 2023; Fivash et al., 2024; Lawrence et al., 2018) will enhance drainage and thereby sediment erosion resistance. Stoorvogel, Temmerman, et al. (2024) showed that locations closer to creeks were more consolidated and had a higher shear strength at the sediment surface, while Fivash et al. (2024) found that sediment on ridges dried out faster than sediment in runnels, leading to higher erosion resistance on the ridges.

### Vegetation establishment and growth

Vegetation establishment requires the availability of propagules, such as seeds, rhizomes, or corms, and disturbance‐free “windows of opportunity” for the propagules to germinate and anchor in the sediment (Balke et al., 2014; Hu et al., 2015). “Windows of opportunity” are short periods during which the deposited seeds or establishing seedlings are not affected by tidal flooding, strong hydrodynamic forcing, nor strong sediment erosion or accretion events, which could cause seed redistribution or seedling uprooting (Balke et al., 2014; Cao et al., 2018; Fivash et al., 2023; Hu et al., 2015). Additionally, for germinating seeds or young seedlings to establish, they must be protected from herbivores such as ragworms (Paramor & Hughes, 2004; Zhu et al., 2016), crabs (Qian et al., 2021), and geese (Jobe et al., 2022). Vegetation establishment also depends on the location‐specific tidal range and drainage conditions, with pioneer zones generally found at lower elevations relative to mean high water level in areas with higher tidal ranges (Balke et al., 2016; Kirwan & Guntenspergen, 2010) and in better drained areas (Fivash et al., 2023). The presence of tidal creek banks may positively affect species richness in tidal marshes (Sanderson et al., 2000).

As vegetation gets established, a network of roots will start to develop within the marsh sediment bed. Plant roots increase aggregate stability and provide tensile strength, which reinforce the sediment matrix and thereby reduce lateral and vertical erosion (Brooks et al., 2021; Chen et al., 2012; Grabowski et al., 2011; Lo et al., 2017). Generally, a higher root biomass results in higher sediment strength and erosion resistance (Lo et al., 2017; Stoorvogel, van Belzen, et al., 2024; Wang et al., 2017). Plant species with different root system characteristics affect sediment strength and erosion resistance differently (Brooks et al., 2022; Evans et al., 2022; Stoorvogel, de Smit, et al., 2025; Wang et al., 2017), so succession of vegetation towards new species and/or a higher marsh zone will result in changing erosion resistance. However, Feagin et al. (2009) show that in very specific situations, plant roots can also aid in accelerating erosion when water levels and waves interact with marsh fronts. Additionally, while vegetation reduces erosion at the local scale within vegetation patches, water flow and erosion may accelerate at the larger scale around these patches (Bouma et al., 2009; Bouma et al., 2013; Van Wesenbeeck et al., 2007; Vandenbruwaene, Temmerman, et al., 2011). Two important factors that may affect vegetation growth and thereby sediment erosion resistance are bed elevation relative to the tidal range and nutrient loading. An optimum bed elevation for plant productivity exists as bed elevation affects flood frequency, drainage, and soil salinity (Morris, 2007; Morris et al., 2002). The effect of nutrient loading on vegetation growth seems to be strongly dependent on the marsh site. High nutrient loading to tidal marshes can lead to a reduced root biomass and thereby to a reduced resistance of the marsh to erosion (Darby & Turner, 2008; Deegan et al., 2012), as well as to increased herbivory and marsh dieback (Tomasula et al., 2023). However, other studies found that belowground biomass increased with nutrient loading (Morris et al., 2014), or that fertilization may increase plant growth in nutrient‐poor sediments (Broome et al., 1988).

### Biotic interactions

For optimal nature‐based flood risk mitigation it is important to also consider species interactions within tidal marshes and across coastal/estuarine habitat mosaics. Ecosystems that occur lower in the tidal zone, such as seagrass meadows and mussel or oyster reefs, can stabilize and accrete sediments, thereby creating a more favorable environment for higher intertidal ecosystems such as tidal marshes (Crotty et al., 2023; Koppel et al., 2015; Schoonees et al., 2019; Vozzo et al., 2023). Interactions between multiple habitats may contribute to a more resilient system (Schoonees et al., 2019), and the momentum for combining ecosystems at the “seascape” scale is therefore building (Garbutt et al., 2024). There are global differences in the occurrence of different faunal groups and ecosystems (Yando et al., 2023), making species interactions location dependent and creating a need for local knowledge to appropriately guide the use of ecosystems in restoration designs.

Many interactions between species exist in tidal marshes that may affect sediment erosion resistance (Renzi et al., 2019). For example, vegetation diversity boosts erosion resistance, by providing a more structurally diverse root network for binding sediments (Ford et al., 2016). The presence of mussels positively affects cordgrass (a common tidal marsh grass species) survival during droughts, resulting from enhanced water storage and reduced soil salinity stress (Angelini et al., 2016). Mussels may also actively enhance accretion within tidal wetlands through their suspension feeding and bio‐deposition activities (e.g., Crotty et al., 2023). Another example is the positive effect of the presence of keystone top predators, such as sea otters, on belowground biomass of marsh plants, resulting from sea otters feeding on root‐grazing shore crabs (Hughes et al., 2024). When sea otters re‐entered the food web, after hundreds years of absence, erosion rates in the system declined significantly. Such positive interactions result in healthy, erosion‐resistant ecosystems. As opposed to these positive species interactions, negative species interactions also exist (He et al., 2025). For example, grazing by snails (Smith et al., 2024), crabs (Qian et al., 2021), and geese (Jefferies & Rockwell, 2002), as well as the presence of fungal pathogens (Elmer, 2016), could lead to marsh vegetation dieback. Thus, without effectively functioning food webs tidal marshes may retreat or underperform. It is therefore essential to incorporate these interactions into marsh restoration and creation designs (Renzi et al., 2019; Silliman et al., 2018) and expand the concept and intervention of trophic rewilding in salt marsh restoration (Schmitz et al., 2023).

### Estuary‐ and global‐scale processes

For the successful restoration or creation of tidal marshes it is important to also consider processes operating at larger scales. For marshes to be resilient in the long term, sediment supply and deposition, as well as the width of the intertidal zone, must be sufficient to keep pace with sea‐level rise. If sediment supply and deposition are insufficient, the tidal marsh might drown (Schuerch et al., 2018) and the adjacent tidal flats essential for marsh expansion are likely to disappear (Bouma et al., 2016; Grandjean et al., 2024; Hu et al., 2015). Coastal squeeze may lead to too small accommodation space and width of the intertidal zone, which may result in erosion risks (Doody, 2013; Townend et al., 2011). It is therefore crucial that a wide intertidal zone is available for marsh restoration and creation, which may especially be problematic in microtidal environments as there is less effective space for marshes (e.g., Kirwan et al., 2010; Raposa et al., 2016). Additionally, climate change effects such as sea‐level rise and changes in the frequency and intensity of droughts and storms may directly affect sediment erosion resistance and the delivery of sediment (Cahoon, 2006; Grabowski et al., 2011), thereby affecting the resilience of restored or created marshes over the long term.

## MARSH RESTORATION AND CREATION AT CONTRASTING LOCATIONS, POSSIBLE INTERVENTIONS, AND THEIR EFFECT ON THE DEVELOPMENT RATE OF EROSION RESISTANCE

**FIGURE 1:**
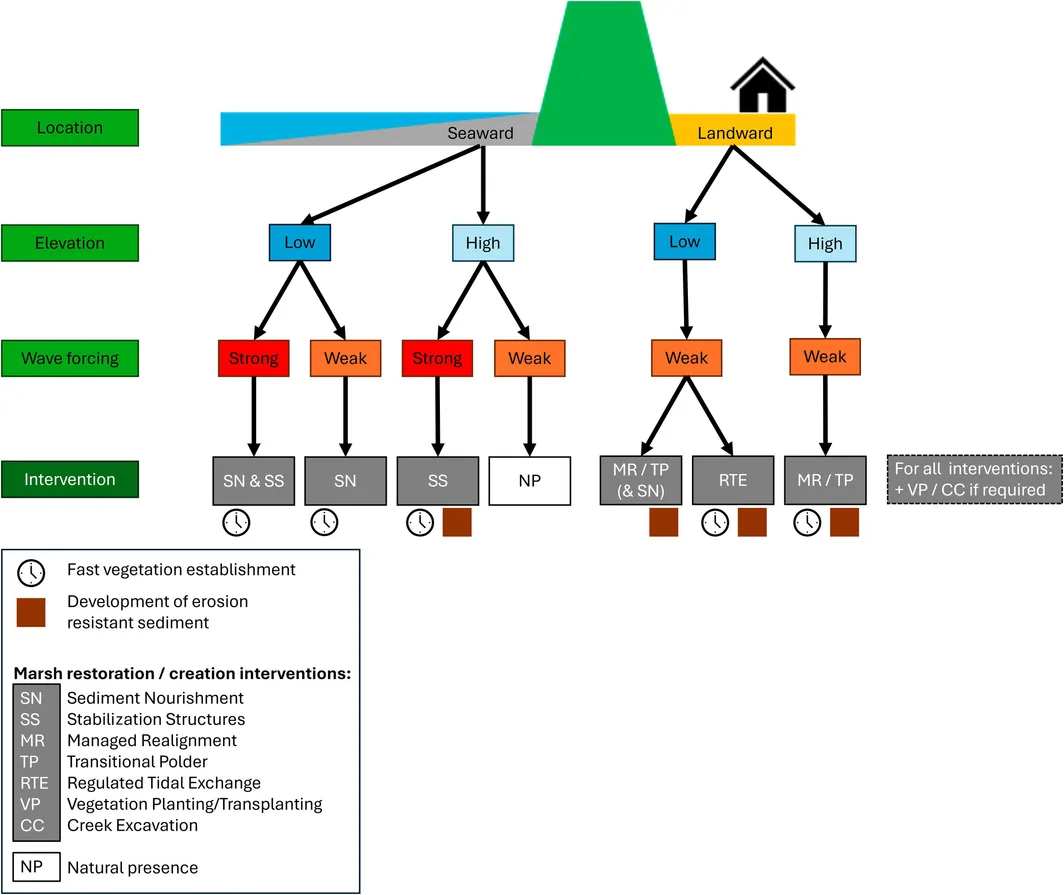
Decision tree based on the three key conditions (location with respect to the existing main defense structure, elevation, and wave forcing) that affect the most suitable marsh restoration or creation intervention for a specific location. In the gray boxes the most suitable marsh restoration and creation interventions are indicated. The white box indicates a situation where it is expected that natural marshes are present, so marsh restoration/creation will not be necessary. Below the gray boxes, at the bottom of the decision tree, we indicate whether fast vegetation establishment and the development of erosion resistant sediment are expected to occur under that intervention. If vegetation establishment or drainage are slow, these can be sped up by applying vegetation planting/transplanting or creek excavation in combination with any of the other interventions (dashed gray box on the right).

The most suitable marsh restoration or creation intervention for flood risk mitigation will largely depend on three key conditions (Figure 1). First, marsh restoration and creation require space in which marshes can develop. At locations where defense structures are used for flood protection, marshes can be restored or created either in front of the existing main defense structure (seaward), or behind the existing main defense structure (landward), with the best option depending on the available, suitable space (Temmerman et al., 2013; Zhu, Vuik, et al., 2020). We here mainly focus on marshes as part of a nature‐based/hybrid coastal protection, using a defense structure as the marker of the terrestrial–marine/estuarine boundary, given that (i) land behind defense structures tends to be lower in elevation and thus at a higher flood risk and with greater potential for siltation following re‐inundation, and (ii) global marsh restoration via the discussed techniques (e.g., managed realignment) is most broadly occurring at such sites. Nonetheless, salt marsh restoration or creation can also take place at locations without defense structures, where the terrestrial–marine/estuarine boundary instead refers to the point beyond which there is no tidal influence, often backed by agricultural land or coastal communities. In that case, this paper refers to “seaward of the defense structure” for locations with tidal influence (seaward of the boundary), while it refers to “landward of the defense structure” for locations without tidal influence (landward of the boundary). Second, the most suitable intervention depends on the initial elevation relative to the tidal range of the location, as this leads to specific tidal inundation characteristics (e.g., inundation frequency, duration, and depth) which are strong determinants of seedling establishment (Balke et al., 2016; Bouma et al., 2016) and further marsh habitat development (Olff et al., 1997). Here, we refer to a low elevation where surface elevation is below the elevation range where tidal marsh vegetation can establish, while a high elevation is within that range. Third and last, the most suitable intervention depends on the strength of hydrodynamic forcing caused by wave action, tidal currents, and storm surges (Möller & Christie, 2018), which will strongly affect the resulting local sediment dynamics and thereby marsh establishment (Bouma et al., 2016; Cao et al., 2018; Cao et al., 2020; Willemsen et al., 2018). This paper mainly focuses on wave forcing as a key hydrodynamic determinant of the most suitable marsh restoration or creation intervention (Figure 1), but we also discuss the effects of tidal range, current forcing, and boat wake forcing, given their well‐established influence on marsh processes and stability (Bilkovic et al., 2019; Friedrichs & Perry, 2001; Kirwan & Guntenspergen, 2010). We refer to a strong wave forcing when the average significant wave height is above 0.2 m and to a weak wave forcing when it is below 0.2 m, based on significant wave heights discussed or measured by Yang et al. (2012), Hu et al. (2021), Hudson et al. (2023), and Willemsen et al. (2018), for example.

### Seaward of the defense structure—low elevation—strong wave forcing

#### Suitable interventions: Sediment nourishment and stabilization structures combined

**FIGURE 2:**
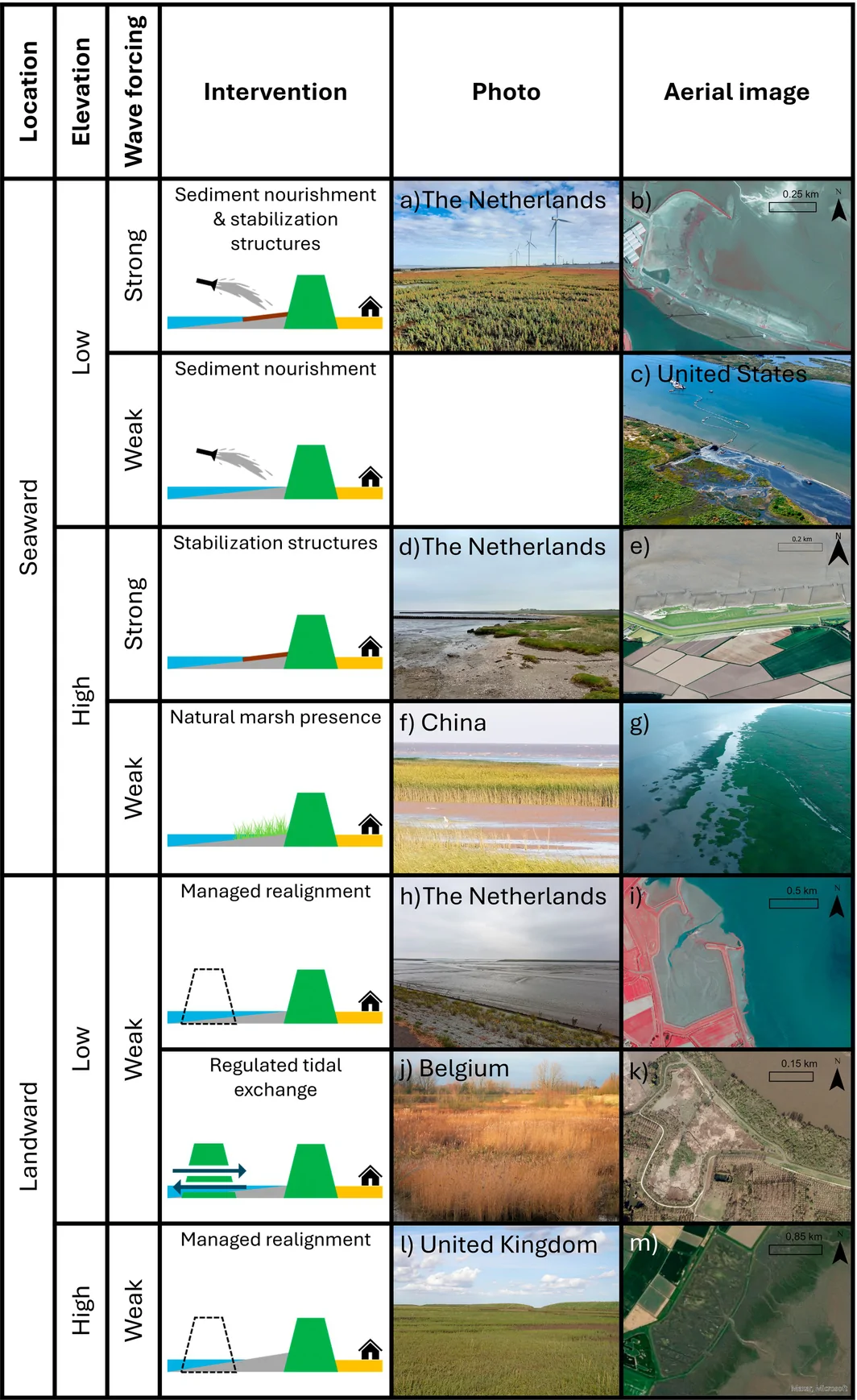
Photographs and aerial images of example sites of the most suitable marsh restoration or creation interventions for different locations with different elevation and wave forcing. (a) Photograph of the created marsh of the Marconi Project near Delfzijl, The Netherlands, where sediment nourishment and stabilization structures were combined (taken by Marte Stoorvogel in 2023). (b) False color aerial image (collected by Rijkswaterstaat in 2020) of the created marsh of the Marconi Project, with red colors indicating vegetation. (c) Photograph of a cutterhead dredge removing shoal material from the Mississippi River, US, during navigation channel maintenance. Fluidized dredged material transported via pipeline spreads as a thin layer along the river's bank to fill shallow depressions and nourish existing wetland plants (photo courtesy of the U.S. Army Corps of Engineers, taken in 2022). (d) Photograph of the marsh at Wierum, The Netherlands, where brushwood groynes were installed to prevent marsh cliff erosion (taken by Victoria Mason in 2024). (e) True color aerial image (created by Sarah Dzimballa, collected by Eelke Folmer of Aeria in 2024) of the marsh at Wierum. (f) Photograph of the Chongming Dongtan wetland, China (taken by Haobing Cao in 2024), which is a natural tidal marsh. (g) Aerial image (collected by Haobing Cao in 2023) of the Chongming Dongtan wetland. (h) Photograph of the restored marsh of Perkpolder, The Netherlands, where managed realignment was applied (taken by Marte Stoorvogel in 2021). (i) False color aerial image (collected by Rijkswaterstaat in 2022) of the restored marsh of Perkpolder, with red colors indicating vegetation. (j) Photograph of the restored marsh of Lippenbroek, Belgium, where regulated tidal exchange (controlled reduced tide) was applied (taken by Lotte Oosterlee in 2012). (k) True color aerial image (collected by “Digitaal Vlaanderen” in 2020) of the restored marsh of Lippenbroek. (l) Photograph of the restored marsh of Freiston Shore, UK, where managed realignment was applied (taken by Iris Möller in 2006). (m) True color satellite image (source: Esri, Maxar, Earthstar Geographics, and the GIS User Community) of the restored marsh at Freiston Shore.

At locations seaward of the defense structure (or seaward of the terrestrial–marine/estuarine boundary at sites without defense structures) with low elevation and strong wave forcing, vegetation establishment and marsh development may be hampered by high inundation frequency, long inundation duration, and high bed shear stresses causing sediment dynamics that uproot seedlings (Balke et al., 2014; Balke et al., 2016; Bouma et al., 2016; Hu et al., 2015). Sediment nourishment, that is, the artificial increase of surface elevation to an elevation that is suitable for marsh development (de Groot & Van Duin, 2013; Hudson et al., 2023; Vuik et al., 2019), and the implementation of stabilization structures to reduce wave energy or stabilize sediments where the foreshore space is not sufficiently wide (de Groot & Van Duin, 2013; Hudson et al., 2023; Vuik et al., 2019) could improve conditions for vegetation establishment and marsh development (Figure 1). The Marconi Project in The Netherlands (Baptist et al., 2021; Figure 2a,b) and Seal Beach National Wildlife Refuge in the United States (Thorne et al., 2019) are examples of locations where sediment nourishment and stabilization structures were combined for tidal marsh creation.

Sediment nourishment (sometimes referred to as intertidal recharge) is often done with (dredged) sandy sediments, but clay and silt sediments can be mixed through the sand (as e.g., done at the Marconi Project, The Netherlands) or placed as a layer on top of the sand bed (Baptist et al., 2021; Vuik et al., 2019). Sediment nourishment can either refer to the creation of new marshes via sediment application where these do not yet occur (e.g., Baptist et al., 2021; Cornwell et al., 2020), or to the restoration of existing, degrading marshes via the application of thin layers of sediment, called thin layer placement (e.g., Thorne et al., 2019; VanZomeren et al., 2018; Figure 2c). Vegetation establishment is typically fast (Figure 1), as raising the bed surface elevation creates “windows of opportunity” for vegetation establishment (Balke et al., 2014). Additionally, plant production may be increased or decreased due to the nourishment, depending on whether the initial elevation lay below or above the optimum marsh elevation for plants productivity (Morris, 2007; Morris et al., 2002). It is crucial to increase the elevation to a level that matches the elevation niche of targeted tidal marsh species, which requires prior knowledge of elevation niches. If the nourished sediments are sandy, sparsely growing annual vegetation types, such as *Salicornia* spp., are most likely to establish (Bouma et al., 2013; Langlois et al., 2003). Such sparsely growing but fast colonizing marsh plants tend to create a less channeled marsh landscape (Schwarz et al., 2018), which is less favorable for sediment pore water drainage, sediment consolidation, sediment accretion, and long‐term marsh resilience to sea‐level rise (Bouma et al., 2007; Schwarz et al., 2018). Sediment accretion is not only important for keeping pace with sea‐level rise, but also to allow fine sediment deposition on sandy nourishments (Fagherazzi et al., 2012). The marsh sediment type may affect not only establishing vegetation species but also the chance of vegetation survival (Crawford & Stone, 2015). The amount of sediment that will be nourished should be carefully weighed from a physical, ecological, and financial perspective and will be site‐dependent. If design requirements for nourished sediments allow for some elevational heterogeneity, this may accelerate vegetation establishment by stimulating local‐scale drainage (Fivash et al., 2023), while generally reducing construction costs.

Stabilization structures such as groynes can lower hydrodynamic forcing and thereby prevent or reduce sediment erosion and enhance sedimentation (de Groot & Van Duin, 2013; Grandjean et al., 2023; Hudson et al., 2023; Vuik et al., 2019). Stabilization structures can consist of gray materials (e.g., stone or concrete) resulting in hard engineering, or of natural materials (e.g., brushwood) or even living reefs (e.g., mussel or oyster reefs), resulting in softer approaches (Schoonees et al., 2019). If sediment nourishment and stabilization structures are combined, the structures can stabilize the nourished sediment (Baptist et al., 2021; de Groot & Van Duin, 2013), which is especially essential when there is not sufficient space available for the development of a typical foreshore and erosion risks are therefore high. The potential for local scour around the structures should however be assessed, especially at locations under high hydrodynamic forcing and with sandy sediment (Winterwerp et al., 2020). The suitability of artificial structures should additionally be considered within the tidal context of the restoration site. In a microtidal system, the ability of the saltmarsh to accrete is often dependent on the supply of sediment during storm‐surge events (Friedrichs & Perry, 2001; Zhu & Wiberg, 2022). As such, placing structures that may reduce sediment delivery during storm surges may impact the ability of the marsh to keep pace with sea‐level rise and consequently reduce the sustainability of the restored marsh as a flood risk mitigation solution. The reduction in hydrodynamic forcing is expected to positively influence vegetation establishment (Figure 1), as seedling uprooting and burial probabilities will be reduced (Bouma et al., 2016; Cao et al., 2018; Hu et al., 2015). While all types of groynes are able to reduce waves, project sustainability will benefit from the use of natural materials (e.g., brushwood groynes or living mussel or oyster reefs) instead of hard engineering constructions (e.g., stone or concrete groynes), as hard engineering constructions may negatively impact morphodynamics and local species (Gittman et al., 2016; Schoonees et al., 2019), with negative implications for marsh flood risk mitigation. Projects should ideally aim for a sequence of adjacent, natural ecosystems, for example, from mussel or oyster reefs low in the intertidal zone, over seagrass meadows at a higher elevation in the intertidal zone, up to marshes highest in the intertidal zone, as illustrated in fig. 5 in Schoonees et al. (2019), as increasingly proposed for the landscape or “seascape” scale (Gillis et al., 2017; Koppel et al., 2015; Vozzo et al., 2023).

#### Sediment nourishment and stabilization structures combined: Development of erosion resistance

Sandy nourishments will have high sand fractions and therefore sediment erosion resistance might be low (e.g., Lo et al., 2017; Marin‐Diaz et al., 2022; Figure 1). Erosion resistance can be increased in two ways: by mixing cohesive clays and/or silts through the sand or by covering the sand with a top layer of clay or silt (Baptist et al., 2021; Grabowski et al., 2011; Vuik et al., 2019). When mixing cohesive sediments through sand, it can be difficult to achieve a homogeneous mix (Baptist et al., 2021; Stoorvogel, Willemsen, et al., 2025), so this method should be improved to optimally increase erosion resistance of the resulting sediment. If sand is overlain by a too thin top layer of clay or silt, the formation of cracks in the fine‐grained surface layer can extend downward into the underlying sandy sediment, potentially triggering erosion of the entire sediment bed (Marin‐Diaz et al., 2022). Sediment that accretes in between the stabilization structures may be fine and cohesive, which will increase erosion resistance (Grabowski et al., 2011).

The presence of vegetation will increase erosion resistance (Chen et al., 2012; Ford et al., 2016; Lo et al., 2017; Wang et al., 2017), but if the vegetation is sparsely growing (as a result of the sandy sediment) this increase will be more limited (Stoorvogel, Willemsen, et al., 2025). If seed availability (of denser species) is problematic for marsh development, seeding or planting (of denser species) could be considered (Baptist et al., 2021; Hudson et al., 2023; Liu et al., 2024). When considering planting, success rates can be increased by using smart planting strategies such as planting vegetation in clusters rather than in uniform grids (Silliman et al., 2015), with planting density dependent on site hydrodynamic energy (Duggan‐Edwards et al., 2020), or transplanting within artificial structures that mimic the emergent traits that are present in established stands of vegetation (e.g., how the canopies of marsh vegetation reduce near‐bed flow velocity within the vegetation stand and increase sediment stability via their root networks; Liu et al., 2024; Temmink et al., 2020). The approach should ideally always follow bet‐hedging planting designs, where planting is done heterogeneously using different techniques and strategies to spread risk over diverse options (Doherty & Zedler, 2015; Fivash et al., 2022). Planting diverse vegetation communities is also likely to stimulate erosion resistance (Ford et al., 2016). The presence of stabilization structures can also positively affect vegetation establishment due to reduced hydrodynamic forcing (Hu et al., 2015), and thereby decrease erosion rates. Finally, vegetation establishment success could be strongly impacted by biotic factors, such as herbivores, predators, and mutualists (Derksen‐Hooijberg et al., 2018; Qian et al., 2021; Xu et al., 2023), so reintroducing predators (Li et al., 2023; Wu & He, 2024) and mutualists (Derksen‐Hooijberg et al., 2018), simulating predator cues (Li et al., 2023; Xu et al., 2023), or using fencing or hazing to keep herbivores out (Jobe et al., 2022) could improve vegetation establishment.

**FIGURE 3:**
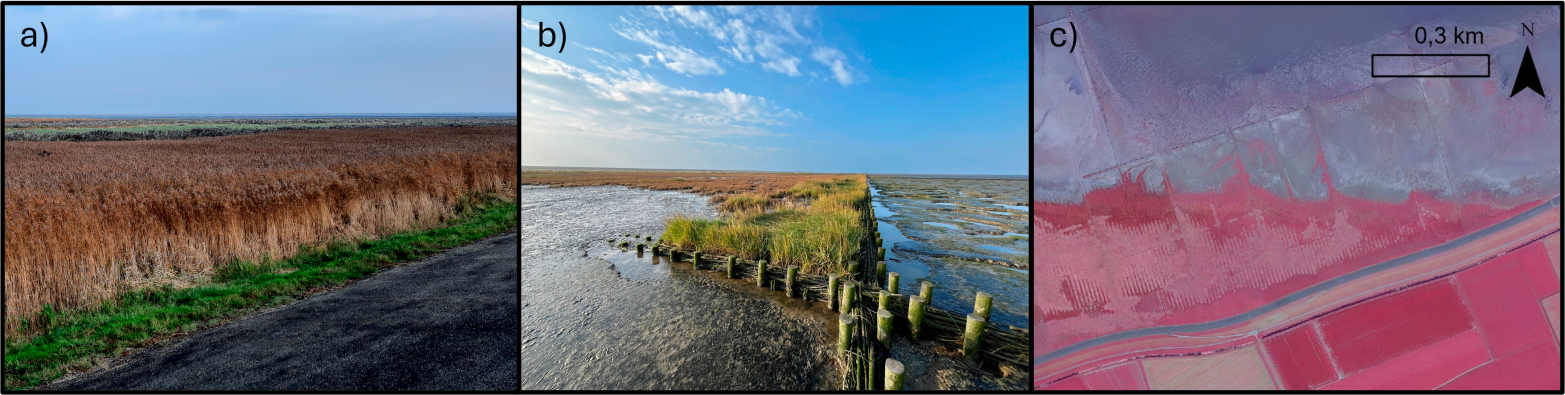
Salt marsh works near Holwerd in the Dutch Wadden Sea. (a) The high tidal marsh that developed through the salt marsh works near the defense structure (taken by Pim Willemsen in 2024). (b) The salt marsh works at the Wadden Sea side of the marsh with *Spartina anglica* and *Salicornia* spp. growing behind the brushwood groynes (taken by Victoria Mason in 2024). (c) False color aerial image (collected by Rijkswaterstaat in 2014) of salt marsh works near Holwerd, with red colors indicating vegetation.

An alternative approach to active sediment nourishment is the construction of sedimentation fields seaward of the defense structure, also called salt marsh works (Bakker, 2014; Bakker et al., 2002; Van Duin et al., 2007; Winterwerp et al., 2020; Figure 3). These sedimentation fields have traditionally been applied at large scale in the Wadden Sea in The Netherlands, Germany, and Denmark to gain habitable and agricultural land (Bakker et al., 2002). This approach entails the construction of sedimentation basins that are surrounded by permeable brushwood groynes, consisting of vertical poles with brushwood placed horizontally in between (Figure 3). Additionally, a dense network of drainage ditches is often dug out to enhance sediment consolidation (Bakker, 2014; Winterwerp et al., 2020). Obviously, reaching a suitable elevation for marsh development will take longer than with active sediment nourishment, but the use of sedimentation fields has successfully led to vegetation and marsh establishment along the Wadden Sea shorelines in the past (Bakker, 2014; Winterwerp et al., 2020). The advantage of sedimentation fields over active sediment nourishment is that the deposited sediment will be silty and hence cohesive and highly erosion resistant (Houwing, 1999; Marin‐Diaz et al., 2022; Winterwerp et al., 2020). The key caveat to this approach is that it may lead to deepening of the shore seaward of the sedimentation fields, which may lead to increased erosion risks. The local site‐specific conditions should therefore be assessed prior to starting this intervention to make predictions about the long‐term sustainability.

### Seaward of the defense structure—low elevation—weak wave forcing

#### Suitable interventions: Sediment nourishment

At locations seaward of the defense structure (or seaward of the terrestrial–marine/estuarine boundary at sites without defense structures) with low elevation but with weak wave forcing, sediment nourishment (as explained in Section: *Suitable interventions: sediment nourishment and stabilization structures combined*) could be a suitable intervention to artificially increase surface elevation if there is not sufficient time to wait for marsh development through natural sedimentation processes (Figure 1). Such sediment nourishment has been applied in areas such as West Bay, Louisiana (Suedel et al., 2022), and in Avalon, New Jersey (VanZomeren et al., 2018). If wave forcing is naturally relatively weak, the addition of stabilization structures may not be necessary to preserve the sediment at nourishment sites. Instead of nourishing sediment directly onto the marsh, sediment could alternatively be disposed in the vicinity of the marsh, for example, in a tidal channel. From here, the sediment could be dispersed towards nearby mudflats and tidal marshes by natural processes (i.e., tidal currents, waves), as was for example done in the Mud Motor in the Dutch Wadden Sea (Baptist et al., 2019). The weaker wave forcing is expected to increase vegetation establishment, as the probability that seedlings are uprooted is smaller (Hu et al., 2015, 2021; Figure 1). It is however important to also consider tidal range, as tidal range can impact the elevation where vegetation can occur and the likelihood of marsh persistence under sea‐level rise (Balke et al., 2016; Fagherazzi et al., 2012). Additionally, the elevation of the nourishment should correspond with the elevation niche of targeted tidal marsh species, as also discussed in Section: *Suitable interventions: sediment nourishment and stabilization structures combined*. On the other hand, herbivory by ragworms, crabs, geese, and others could limit vegetation establishment (Jobe et al., 2022; Paramor & Hughes, 2004; Qian et al., 2021; Xu et al., 2023), so both abiotic and biotic interactions should be considered at the marsh restoration/creation location.

#### Sediment nourishment: Development of erosion resistance

If sediment nourishments are done with sandy, non‐cohesive sediment, erosion resistance is expected to be relatively low (e.g., Lo et al., 2017; Marin‐Diaz et al., 2022; Figure 1). There are several ways to increase erosion resistance of sediment nourishments as explained in Section: *Sediment nourishment and stabilization structures combined: Development of erosion resistance*. However, the fact that erosion resistance might be relatively low may not be problematic if wave forcing is weak, which will likely lead to low erosion rates anyway (Winterwerp & Van Kesteren, 2004). For the long‐term safety contribution, however, the system needs to gain erosion resistance, to not collapse under rare storm‐wave attacks and to even resist the high flow velocities over the marsh in the case that a defense structure behind the marsh would breach (as explained in Sections: *Introduction* and *Sediment nourishment and stabilization structures combined: Development of erosion resistance*).

### Seaward of the defense structure—high elevation—strong wave forcing

#### Suitable interventions: Stabilization structures

If surface elevation seaward of the defense structure (or seaward of the terrestrial–marine/estuarine boundary at sites without defense structures) is naturally relatively high but wave forcing is too strong for vegetation to establish, the implementation of wave‐attenuating stabilization structures (as explained in Section: *Suitable interventions: sediment nourishment and stabilization structures combined*) may enable vegetation and marsh establishment by creating “windows of opportunity” (Cao et al., 2018; Hu et al., 2015) and at the same time contribute to long‐term erosion protection (Garbutt et al., 2006; Grandjean et al., 2023; Hudson et al., 2023; Figure 1). An example of such a site is Wierum in The Netherlands, where new brushwood structures were installed in 2024 to prevent marsh cliff erosion (RTV Noordoost Friesland, 2024; Figure 2d,e). Other locations where stabilization structures have been applied for marsh restoration at preexisting marshes are the Venice Lagoon in Italy (Scarton et al., 2000) and the Severn Estuary in the United Kingdom (Dale & Farrell, 2026; Natural Resources Wales, 2025) using brushwood structures, while at Abbotts Hall Farm in the United Kingdom they installed coir roles in creeks (Essex Wildlife Trust, 2018). Similarly, on the east coast of the United States a “living shorelines” approach has been employed, utilizing permeable breakwaters to reduce marsh lateral erosion (Polk et al., 2021; Smith et al., 2018). An alternative way to overcome establishment thresholds that cause seedling loss might be smart planting strategies, as explained in Section: *Sediment nourishment and stabilization structures combined: Development of erosion resistance*. Such approaches will however only be successful if vegetation patches are large/dense enough to maintain themselves by mitigating physical stress. In cases where vegetation patches are not self‐sustaining and thus a purely “green” solution is not feasible, such measures could be combined with more permanent engineering approaches (Temmink et al., 2023).

#### Stabilization strcutures: Development of erosion resistance

The sheltered conditions in between stabilization structures are expected to have two main effects. First, sedimentation rates will likely increase (Hudson et al., 2023; Vuik et al., 2019), although the implementation of structures in microtidal systems may reduce sediment delivery during storm surges (Friedrichs & Perry, 2001; Zhu & Wiberg, 2022). If accreting sediments are fine and cohesive, they will increase erosion resistance (Grabowski et al., 2011; Figure 1). Second, erosion will decrease, which leads to more suitable conditions for vegetation establishment (Cao et al., 2018). In turn, the plant roots will enhance erosion resistance (e.g., Lo et al., 2017; Wang et al., 2017). On a local scale, however, the presence of stabilization structures may lead to scour around the structures, which should be assessed to determine whether to take measures to prevent this from happening (Winterwerp et al., 2020).

### Seaward of the defense structure—high elevation—weak wave forcing

#### Suitable interventions

There is a high probability that tidal marshes already exist at locations with relatively high elevation and weak wave forcing, for example at the Chongming Dongtan wetland in the Yangtze Estuary in China (Ge et al., 2016; Hu et al., 2021; Figure 2f,g), as there will be sufficient “windows of opportunity” and small enough sediment disturbances for seedling establishment (Balke et al., 2016; Cao et al., 2018; Hu et al., 2015; Figure 1). If no marshes exist at locations with relatively high elevation and weak wave forcing, it might be because tidal currents or boat wakes lead to high bed shear stresses (Bilkovic et al., 2019; Hu et al., 2015) or because seed availability is too low, in which case seeding or planting of marsh species could be considered (Baptist et al., 2021; Hudson et al., 2023; Liu et al., 2024). As described in Section: *Sediment nourishment and stabilization structures combined: Development of erosion resistance*, planting may benefit from smart planting strategies. Other reasons for the absence of marshes at such locations could be poor drainage, unstable sediments, and high sedimentation rates (resulting in poor drainage and consolidation; Cao et al., 2018, 2021; Stoorvogel, Temmerman, et al., 2024). Lastly, marsh establishment may be inhibited by too much bioturbation or grazing by crabs, worms, snails, or geese (Jobe et al., 2022; Qian et al., 2021; Xu et al., 2023). Reintroducing predators (Li et al., 2023; Wu & He, 2024) and mutualists (Derksen‐Hooijberg et al., 2018), simulating predator cues (Li et al., 2023; Xu et al., 2023), or using fencing or hazing to keep herbivores out (Jobe et al., 2022) may improve marsh establishment.

#### Natural marshes: Development of erosion resistance

Sediment beds of mature climax marshes are generally very erosion resistant due to the consolidated, cohesive sediments and plant roots (Marin‐Diaz et al., 2022; Schoutens et al., 2022). However, younger pioneer marshes might be more vulnerable to erosion and require time to further develop erosion resistance (Marin‐Diaz et al., 2022; Stoorvogel, Willemsen, et al., 2025). If there is an aim to use naturally developing tidal marshes for nature‐based flood risk mitigation, it is important to consider the age of the tidal marsh and potentially test its erosion resistance (e.g., with methods discussed in Brooks et al., 2022, 2023; Stoorvogel, Temmerman, et al., 2024) in order to gain insight into the contribution of the marsh to long‐term nature‐based flood risk mitigation.

### Landward of the defense structure—low elevation—weak wave forcing

#### Suitable interventions: Managed realignment/transitional polder, regulated tidal exchange

Where defense structures are present, a site landward of the defense structure at flat locations (e.g., The Netherlands) often has a relatively low surface elevation, resulting from land subsidence following historic land reclamation (e.g., due to soil drainage and groundwater extraction) and a lack of sediment supply, deposition, and elevation gain (Syvitski et al., 2009; Weisscher et al., 2022). Wave forcing is often relatively weak when marshes are restored landward of a defense structure, especially when the area is relatively small and remains (partly) protected by remaining surrounding defense structures (Morris, 2013; Wiesebron et al., 2024). Interventions to restore or create tidal marshes where they currently do not occur landward of the defense structure where elevation is low are: (1) managed realignment, (2) transitional polders, and (3) regulated tidal exchange (all interventions are explained below; Figure 1). Depending on the amount of time before the marshes are necessary for nature‐based flood risk mitigation and on the desired rates of sediment accretion, vegetation establishment, and development of sediment erosion resistance, some interventions are more suitable than others.

In managed realignment, an existing defense structure is removed, breached, or lowered to reintroduce the tides in a previously embanked area (French, 2006; Schuerch et al., 2022), as was, for example, done at Perkpolder in The Netherlands (Walles et al., 2019; Figure 2h,i) and Tollesbury in the United Kingdom (ABPmer, 2024; Garbutt et al., 2006; McMahon et al., 2023). At the landward side of the area where tides are reintroduced, upsloping topography, existing secondary defense structures, or newly built defense structures become the primary flood defense line (Hudson et al., 2023; van den Hoven et al., 2022). While managed realignment sites remain permanently open to the tides, a temporary controlled dike breach is made for transitional polders between double dikes (Van Belzen et al., 2021; Weisscher et al., 2022; Zhu, Vuik, et al., 2020). The site will first transform into a tidal flat, where sediment accretion and surface elevation gain will take place, which favors the development into a higher elevated, vegetated tidal marsh. After sufficient surface level rise, the dike breach can be closed again and the tidal marsh can be converted back to human land use such as agriculture (Van Belzen et al., 2021; Weisscher et al., 2022; Zhu, Vuik, et al., 2020). This intervention can be applied in cases where it is not desirable to convert the agricultural land permanently into tidal marshes, but rather where the goal is to stimulate land accretion for future human usage (Weisscher et al., 2022; Zhu, Vuik, et al., 2020). A first pilot project of a transitional polder in between double dikes is currently being created near Bierum, The Netherlands (Eems‐Dollard 2050, n.d.). Managed realignment and transitional polder sites with low elevations will likely experience high initial sedimentation rates if suspended sediment concentrations are sufficient, due to the high tidal inundation frequency, duration, and depth and large accommodation space (i.e., the space available for sediment and organic matter accumulation; Gore et al., 2024; Oosterlee et al., 2020; Rogers et al., 2019; Temmerman et al., 2004). The relatively calm conditions are expected to promote the sedimentation of fine sediment. The high inundation frequency will initially limit “windows of opportunity” for vegetation establishment (Balke et al., 2016; Fivash et al., 2023), thereby preventing or slowing down marsh development (Figure 1). Additionally, sediment drainage can be very poor because of (1) the low elevation (small hydraulic gradient towards the estuary at low tides) and (2) the high sedimentation rate, which may together lead to poorly consolidated, very weak, almost fluid‐like sediment beds (e.g., at Burchtse Weel in the Scheldt estuary in Belgium; Oosterlee et al., 2020). Together, the lack of “windows of opportunity” and poor drainage may limit the colonization of plants and benthic invertebrates (Cao et al., 2021; Oosterlee et al., 2020). Sediment nourishment could be applied in such managed realignment and transitional polder areas to speed up vegetation establishment and marsh development (French, 2006). Providing drainage networks in such areas may also accelerate marsh development (for details see end of Section: *Managed realignment/transitional polder, regulated tidal exchange: Development of erosion resistance*). It should always be assessed whether the tidal range is suitable for vegetation establishment (Balke et al., 2016): Perkpolder in The Netherlands (Walles et al., 2019; Figure 2h,i) and Tollesbury in the United Kingdom (ABPmer, 2024; Garbutt et al., 2006; McMahon et al., 2023) are managed realignment projects in estuaries at locations with low surface elevation, where sediment accretion is needed before a marsh may develop. Although the majority of managed realignment sites occur in estuaries, managed realignment can also be applied at the open coast, as for example at Medmerry in the United Kingdom (Dale et al., 2018; Williams & Dale, 2023). The wave climate, tidal forcing, and sediment availability will likely be different at such managed realignments at the open coast than in estuaries, which is important to consider as this may strongly affect marsh development, for example, local wave height and sediment supply were shown to be strong determinants of seedling establishment (Hu et al., 2021).

Regulated tidal exchange is similar to managed realignment, but the original defense structure stays in place, and culverts or (self‐regulating) tide gates are installed in the defense structure to regulate the tidal exchange between the sea/estuary and the marsh restoration site, to enable full control over the tidal regime in the restoration site (Hudson et al., 2023; Jacobs et al., 2009; Maris et al., 2007; Masselink et al., 2017). Examples of such sites are Lippenbroek and other Sigma Plan sites in Belgium (Beauchard et al., 2011; Sigmaplan, 2025; Figure 2j,k) and Goosemoor in the United Kingdom (ABPmer, 2024). An important benefit of regulated tidal exchange is that tidal flooding can be reduced compared to full tidal exchange (e.g., managed realignment), which allows for faster vegetation establishment and marsh development at low elevation sites, as it will not be necessary to wait for sufficient sedimentation and elevation gain to occur (Hudson et al., 2023; Oosterlee et al., 2020; Vandenbruwaene, Maris, et al., 2011; Figure 1). A controlled reduced tidal exchange lowers sedimentation rates compared to a full tidal exchange, due to the reduced tidal inundation depth, frequency, and duration (Oosterlee et al., 2020). As the purpose of many regulated tidal exchange sites is to contribute to flood risk mitigation through water storage during floods (as e.g., the case for the Sigma Plan sites; Cox et al., 2006), lower sedimentation rates leading to a higher water storage capacity are beneficial.

Whether a salt marsh is restored or created using managed realignment or regulated tidal exchange, as well as the design of such a site (e.g., the size and number of breaches and creeks), may strongly influence the contribution of a marsh to flood risk mitigation and the development of different habitats (Gourgue et al., 2022; Kiesel et al., 2020; Oosterlee et al., 2020; Schuerch et al., 2022; Stamp et al., 2023). The implemented restoration/creation design should therefore be carefully considered before marsh restoration or creation is started.

#### Managed realignment/transitional polder, regulated tidal exchange: Development of erosion resistance

If accreted sediments at managed realignment, transitional polder, and regulated tidal exchange sites are fine and cohesive, these sediments have the potential to become highly erosion resistant over time (Grabowski et al., 2011; Houwing, 1999; Figure 1). However, at managed realignment and transitional polder sites with low elevation, the accreted sediments can initially be very wet due to frequent and long tidal inundations and slow drainage caused by fine sediments and a strongly consolidated relict agricultural soil underneath the newly deposited sediment that forms an impermeable layer for groundwater (Brooks et al., 2021; Spencer et al., 2017; Van Putte et al., 2020; Van Rees et al., 2024). This may slow down consolidation and the development of erosion resistance (Colosimo et al., 2023; Dong et al., 2020; Van Rees et al., 2024; Figure 1). Additionally, the slow drainage may lead to high salinity, anoxic sediments, and the build‐up of potentially toxic chemicals (Spencer et al., 2017). This, in combination with a lack of “windows of opportunity” and the soft, poorly consolidated sediment, will likely also slow vegetation establishment (Balke et al., 2014; Cao et al., 2021), so initially vegetation cannot enhance erosion resistance. It is therefore important to give the system sufficient time to develop erosion resistance, and thus start the managed realignment or transitional polder scheme long enough before the marsh is needed for nature‐based flood risk mitigation.

The development of sediment strength in regulated tidal exchange areas increases with decreasing inundation frequency and sedimentation rate, as a result of stronger drying‐induced consolidation (Stoorvogel, Temmerman, et al., 2024). The culverts or tide gates at regulated tidal exchange sites allow for control over sedimentation rates and drainage (Oosterlee et al., 2020), which can promote the development of tidal flats or marshes with high erosion resistance. One such option is to use a full tidal exchange at first to allow for very fast sedimentation, after which the culverts can be closed to let the sediment drain and consolidate, thereby increasing the erosion resistance of the sediment (Oosterlee et al., 2020; Figure 1). This can be an advantage over defense structure breaching in managed realignment, where such detailed tide control would not be possible. However, fast sedimentation rates will lead to smaller water storage capacity, so it must be considered whether the fast development of an erosion‐resistant high marsh or large water storage capacity is most desirable, depending on the exact goals and larger scale landscape context of the project (Cox et al., 2006; Oosterlee et al., 2020). Where marshes occur along funnel‐shaped estuaries, water storage on marshes can strongly reduce the upstream peak height of storm surges through lateral spreading of these surges, while the effect on the peak height of storm surges is smaller where marshes occur along, for example, open coasts or bays and wide estuaries (Temmerman et al., 2023; Weisscher et al., 2022).

Digging creeks at managed realignment, transitional polder, and regulated tidal exchange sites may strongly improve drainage (Hudson et al., 2023; Van Putte et al., 2020), and thereby accelerate sediment consolidation and vegetation establishment (Brain, 2015; Cao et al., 2021), resulting in the faster development of erosion resistance. This is why traditional salt marsh works were typically well‐drained (Bakker, 2014; Winterwerp et al., 2020; see Section: *Sediment nourishment and stabilization structures combined: Development of erosion resistance*).

### Landward of the defense structure—high elevation—weak wave forcing

#### Suitable interventions: Managed realignment/transitional polder

A site landward of the defense structure with higher surface elevation does not require the implementation of regulated tidal exchange (e.g., a controlled reduced tidal exchange), as the inundation regime, due to its high elevation, is already suitable for marsh development (Balke et al., 2016). This is, for example, the case at Freiston Shore in the United Kingdom, where a managed realignment project took place at a location with relatively high surface elevation (Morris, 2013; Friess et al., 2012; Figure 2l,m). A higher elevation of managed realignment or transitional polder sites will lead to more “windows of opportunity” for vegetation establishment (also depending on the tidal range; Balke et al., 2014), and thereby faster vegetation establishment and marsh development (Figure 1). The higher surface elevation will likely lead to lower sedimentation rates than at sites with low surface elevation (Oosterlee et al., 2020; Temmerman et al., 2004). If the high surface elevation leads to low natural dynamics and species richness, top‐soil removal may be considered to increase tidal inundation and set back vegetation succession of the site (Elschot et al., 2024).

#### Managed realignment/transitional polder: Development of erosion resistance

The faster vegetation establishment is expected to lead to a faster development of erosion resistance, as roots will reinforce the sediment matrix (e.g., Brooks et al., 2021; Chen et al., 2012; Lo et al., 2017). Additionally, tidal inundation frequency, duration, and depth will be lower and sediment bed drainage faster than with a low elevation, leading to drier sediment that consolidates and becomes stronger faster (Stoorvogel, Temmerman, et al., 2024; Figure 1). At locations without significant sedimentation, sediment erosion resistance will also depend on the former land use before managed realignment (Watts et al., 2003).

## OUTLOOK

Although tidal marshes can strongly contribute to nature‐based flood risk mitigation, marsh incorporation into flood defenses may not be appropriate and successful everywhere. This highlights the importance of considering the characteristics of a location where marsh restoration or creation is planned, not only from a physical and ecological perspective, but also from a social, economic, and governance perspective. Location‐specific factors such as the original land use should be considered, before valuable subtidal areas or agricultural land might be turned into tidal marshes. Additionally, there may be external processes that can strongly affect the effectiveness of marshes for flood risk mitigation in coming years, for instance where larger scale management has stronger effects than site‐scale decisions. For example, if marshes are created within an estuary, this might reduce the water storage capacity, potentially heightening storm‐surge water levels, while marsh creation at the landward side of a seawall could have the opposite effect. While ideally after marsh restoration or creation the marsh becomes self‐sustaining, restored or created wetlands may require adaptive management in which they are actively managed, adjusted, and/or maintained (Elschot et al., 2024; Hudson et al., 2023). Such future management decisions (e.g., the introduction of grazers, mowing, public use) could affect how erosion resistance develops (e.g., Marin‐Diaz et al., 2021). The occurrence of certain ecosystem engineers, predators, and ecosystem types can also strongly affect vegetation health and erosion resistance of tidal marshes (e.g., Angelini et al., 2016; Ford et al., 2016; Hughes et al., 2024) and should therefore be considered in marsh restoration and creation projects.

Although we are gaining increasing insight into the contribution of tidal marshes to nature‐based flood risk mitigation, a better understanding of several mechanisms is still required for the optimal integration of tidal marshes into flood defenses, such as (1) further quantifying the effectiveness of marshes via wave attenuation; (2) understanding the potential impacts of climate‐change driven impacts on marsh health and erosion resistance; and (3) understanding how marshes will interact with hard structures where “gray‐green” flood defense strategies are the most appropriate solution.

The potential for the incorporation of marshes within nature‐based flood risk mitigation strategies is clear; the success of such strategies depends on (1) selecting the most appropriate marsh restoration or creation intervention for a specific location, and (2) giving the marsh enough time to develop before it is needed for flood risk mitigation. The long‐term success of such sites will ultimately depend on the presence of sufficient space over a gently sloping gradient to allow sediment deposition and to prevent erosion. The incorporation of marshes within nature‐based flood risk mitigation strategies should not only be considered at an individual site scale but also at larger landscape scale consequences for flood risk mitigation, to ensure a fully functioning wider ecosystem with good connectivity between ecosystems.

## CONCLUSIONS

Tidal areas, consisting of tidal flats and tidal marshes, can strongly contribute to nature‐based flood risk mitigation. Tidal marshes provide greater benefits than tidal flats because (1) their vegetation and higher elevation within the tidal prism both contribute to wave attenuation, and (2) they have stabilized sediments that can prevent shoreline erosion (Marin‐Diaz et al., 2022; Möller et al., 2014) and reduce the depth of a defense structure breach and consequential flood damage significantly (Van den Hoven et al., 2023; Zhu, Vuik, et al., 2020). Additionally, sediment accretion by marshes can enable them to keep up with sea‐level rise (Coleman et al., 2022). Wave attenuation, especially by tidal marshes, may allow a lower required defense structure height for flood protection, and marsh restoration or creation can globally lead to more affordable and sustainable flood risk mitigation in the long‐term compared to the use of gray flood defense structures (Temmerman et al., 2013; Zelst et al., 2021). This creates potential for the implementation of marsh restoration and creation for nature‐based flood risk mitigation.

Here, we discussed the contrasting locations at which tidal marsh restoration and creation could take place and how the application of different marsh restoration/creation interventions will likely affect the development of sediment erosion resistance at these locations. A restoration or creation intervention should be chosen depending on the available space (seaward or landward of the defense structure), local environmental conditions (e.g., elevation, hydrodynamic forcing by waves and tides, tidal range, grain size distribution of available sediment, seed availability), and the amount of time until the enhanced flood risk mitigation is necessary (Figure 1). Using certain interventions, such as managed realignment, erosion resistance will develop slowly, but erosion resistance is expected to become higher over time compared to other interventions (due to cohesive sediments and dense vegetation; Figure 1). Therefore, the main priority is to select the most appropriate marsh restoration or creation intervention for a specific location and to plan well ahead of time, to give accreting sediment in marshes time to become highly erosion resistant, as observed in mature climax marshes (Marin‐Diaz et al., 2022; Schoutens et al., 2022).

## AUTHOR CONTRIBUTIONS

Marte M. Stoorvogel: Conceptualization, writing—original draft. Bas W. Borsje: Supervision, project administration, funding acquisition. Tjeerd J. Bouma: Conceptualization, supervision, project administration, funding acquisition. All authors contributed to writing the manuscript and reviewed and edited the draft manuscripts.

## CONFLICT OF INTEREST STATEMENT

The authors declare no conflicts of interest.
